# Evaluation of liver enzyme levels in workers exposed to vinyl chloride vapors in a petrochemical complex: a cross-sectional study

**DOI:** 10.1186/1745-6673-2-6

**Published:** 2007-08-08

**Authors:** Mir Saeed Attarchi, Omid Aminian, Mandana Dolati, Maria Mazaheri

**Affiliations:** 1 Department of Occupational Medicine, Faculty of Medicine, Iran University of Medical Sciences, Tehran, Iran; 2 Department of Occupational Medicine, Faculty of Medicine, Tehran University of Medical Sciences, Tehran, Iran; 3 Department of Pathology, Legal Medicine Organization, Tehran, Iran; 4 Environmental & Occupational Health Center, Ministry of Health & Medical Education, Tehran, Iran

## Abstract

**Background:**

Polyvinyl chloride is used in production and manufacturing of many essential tools (e.g. plastic pipes, photography films, etc.). Its production is impossible without the use of vinyl chloride monomer (VCM), which can cause liver damage in long-term. In this study we intend to assess the effects of mild to moderate long term exposure to VCM on liver and to assess the importance of liver enzyme measurements as a screening tool.

**Methods:**

In this study, liver enzyme levels of 52 workers were compared to 48 control workers using the T-test. The cases all worked in a PVC production unit in a petrochemical complex and the controls were randomly selected from office personnel of the same complex. A questionnaire was also filled in about information such as age, weight, work history, etc. in both groups.

**Results:**

Mean comparisons for ALP and GGT using T-test showed statistically significant differences between the two groups. For AST, ALT and bilirubin (total, direct) the mean was higher in the case group but this difference was not statistically significant.

**Discussion:**

This study showed that mild exposure to VCM can cause mild liver cholestasis. So, using cholestasis assessment tests such as ALP and GGT should be considered in periodic assessment of liver function in PVC producing units.

## Background

PVC application is increasing day by day. It is used in the production of plastic pipes, cable and wire coatings, floorings, photography films and also electronic industry [1-3]. VCM is an essential element in its production. Regarding the increase of PVC usage and production, more workers are exposed to vinyl chloride every day. Presently more than 81,000 workers are exposed to VCM [1].

VCM is hepatotoxic and carcinogenic and can cause liver damages such as hepatic fibrosis, hepatic angiosarcoma, hepatocellular carcinoma, portal hypertension, etc.

The mechanism of toxicity and carcinogenicity of VCM is hypertrophy and hyperplasia of hepatocytes and sinusuidal cells, sinusuidal dilatation and destruction, hepatocyte destruction, portal tract fibrosis and binding of chloroethylene oxide (VCM metabolite) to DNA and RNA [4-10].

An important subject in health preservation of workers exposed to VCM is the early detection of is effects. Unfortunately minor liver damages can not be detected through routine screening tests such as aminotransferase measurement and needs more specific tests such as the measurement of serum bile acid levels, liver and spleen sonography and indocyanin clearance test [11].

Several studies have been conducted on detection of early effects of VCM on workers with contradictory results. In addition, the role of confounding factors such as BMI, cigarette smoking and alcohol consumption were not taken into account in lots of these studies. Most of the studies were conducted on above the threshold levels of VCM, while regulatory organizations such as ACGIH reduce the threshold day by day (In 2003, the threshold was reduced from 5 ppm to 1 ppm). This shows that VCM can have detrimental effects on human health even below the threshold level.

In many petrochemical complexes workers are exposed to below the threshold levels of VCM. Regarding the increase in the number of such complexes in our country and the few studies conducted about the problem, we designed this study.

## Methods

This is a cross-sectional analytic study. The study was conducted in a PVC production unit in a petrochemical complex in 2005. In this unit, PVC is produced from VCM by a rather long process.

The case group was 52 male workers who worked in the PVC production unit and the control group was 48 male office workers from the same complex. Having at least 2 years of working experience was the inclusion criteria.

Environmental monitoring showed a concentration of 0.7 ppm for VCM. Sampling tube method was used and its analysis was performed by Gas chromatography.

Environmental sampling was done in several consecutive days and during the whole working days, i.e. 8 hours. Maximum exposure level was 0.85 ppm and the mean exposure level was 0.8 ppm in 8 working hours. Previous years' samplings also showed a maximum environmental exposure level of 0.8 ppm.

So the cases were workers who had long time exposure to less than allowable levels (1 ppm) of VCM.

Demographic data including age, weight height, work experience, alcohol consumption, tobacco smoking, past medical history, drug history, performing heavy exercises, work history including any changes of the job and second job, history of surgery and history of blood transfusion were all gathered through a questionnaire.

Workers with systemic diseases (such as thyroid disease, diabetes, etc.) or active hepatic diseases (based on the results of HBs-Ag, HBc-Ab and HCV-Ab tests), those consuming drugs that could affect liver enzyme levels (such as phenytoin, carbamazepin, estrogen, fibric acid, etc.), workers who had undergone coronary bypass surgery or had received blood in the past 3 months and workers who performed heavy exercises were excluded. HBs-Ag, HBc-Ab and HCV-Ab tests were used to rule out active infectious diseases. Blood samples were also used to assess the following 6 biochemical markers:

1 – Liver transaminases (ALT, AST) which show hepatic necrosis

2 – Liver cholestasis tests (GGT, ALP)

3 – Liver clearance tests (total and conjugated bilirubin)

Lab tests along with the questionnaire data were analyzed with SPSS-10 using the T-test and linear regression analysis.

## Results

All samples were Iranian males so there was no difference between the two groups. In addition, no statistically significant difference was found between the groups for age, job experience and BMI. (Table 1)

**Table 1:** Demographic data of studied groups

	**Case (n = 52)**	**Control (n = 48)**	**P-Value**
**Age**			
Mean (yr)	30.8	30.69	>0.05
Median (yr)	30.5	30.5	
Range (yr)	22–41	23–43	
**Work experience**			
Mean (yr)	8.1	9	>0.05
Median (yr)	7.5	8	
Range (yr)	3–15	2–14	
**BMI**			
Mean (kg/m^2^)	22.07	22.29	>0.05
Median (kg/m^2^)	22	22	
Range (kg/m^2^)	19–24	19–26	

Viral hepatic disease tests were performed for all cases and controls. 11 workers (6 cases and 5 controls) were positive and thus excluded. Alcohol consumption was considered positive even if consumed occasionally. 7% of cases and 8% of controls consumed alcohol. 19 cases (36.5%) and 18 controls (37.5%) smoked tobacco. No statistically significant difference was found between the two groups for alcohol consumption and tobacco smoking and so these two can not be confounding.

Mean comparison between two groups and the related P-values are shown in Table 2. GGT and ALP means where significantly higher in case group (P < 0.001). In other tests the absolute values where higher in the case group, but the difference was not significant.

**Table 2:** Comparison of mean of liver tests in studied groups

**Value**	**Mean in case group**	**Mean in control group**	**P-value**
**Test**			
ALT (u/l)	28.88	27.98	0.1
AST (u/l)	22.87	21.15	0.1
ALP (u/l)	188.83	172.15	<0.001
GGT(u/l)	34.06	26.46	<0.001
T-Bili (mg/d)	0.83	0.82	0.6
D-Bili (mg/d)	0.24	0.24	0.4

For further assessment of the relationship between liver enzyme levels and VCM exposure and for proper control of all confounders we used the linear regression analysis. The results are shown in Tables 3 and 4. As it is clear there was only a statistically significant relationship between VCM exposure and ALP and GGT levels.

**Table 3:** Relationship between ALP level and other variables of the study

	β	P-value
**Variable**		
Constant	172.146	0.000
Exposure	16.912	0.000
Age	0.004	0.936
BMI	0.063	0.163
Alcohol	-0.042	0.351
Cigarette	-0.026	0.57

**Table 4:** Relationship between GGT level and other variables of the study

	β	P-value
**Variable**		
Constant	26.479	0.000
Exposure	7.579	0.000
Age	0.017	0.73
BMI	0.017	0.729
Alcohol	0.007	0.886
Cigarette	-0.056	0.267

Cases were divided to 3 subgroups regarding their job experience. There was no statistically significant difference for BMI, age and the number of alcoholics and smokers among these 3 subgroups; but for GGT and ALP the differences of means were significant.

Liver enzyme levels were compared in each group of cases and controls separately regarding the consumption of alcohol and smoking. Liver enzyme levels were not significantly different between smokers and nonsmokers, and alcoholics and non-alcoholics, in each group.

In fact, the liver enzyme levels were compared between 4 cases who consumed alcohol and 48 cases that didn't. There was no statistically significant difference in any of the liver enzyme levels between these 2 subgroups. (P-value = 0.9)

The liver enzyme levels were also compared between 4 controls who consumed alcohol and 44 controls that didn't. There was no statistically significant difference in any of the liver enzyme levels between these 2 subgroups. (P-value = 0.95)

The same applies for smoking and no statistically significant difference was found in any of the 2 groups of cases or controls. (P-value > 0.05)

The only statistically significant difference was between GGT and ALP (P < 0.05) (Table 5).

**Table 5:** Comparison of liver enzyme levels (ALP, GGT) according to work experience in case group

	ALP(U/L)*	GGT(U/L)*
**Working experience**		
Less than 4 years	181.85	26
4–8 years	188.7	34.03
More than 8 years	192.28	43.5

*P < 0.05

## Discussion

In this cross sectional study, we assessed the changes of routine liver tests in workers exposed to VCM with less than allowable levels (as proposed by ACGIH).

We omitted the effect of confounding factors such as age, sex, weight, alcohol consumption, tobacco smoking, job experience and infectious hepatic diseases (hepatitis B & C) which was not done systematically in previous studies.

In workers with positive viral hepatic disease tests, mean comparison showed higher ALT in the case group (P < 0.05). This shows that VCM exposure can aggravate viral hepatic disorders and further increase liver enzymes. Between these two groups there was no statistically significant difference for factors such as age, tobacco smoking, alcohol consumption and job experience, which is in accordance with previous studies[12].

Maybe the increase of sample size would lead to significant difference in the level of other liver enzymes between the 2 groups. But we must take into consideration that in chronic liver diseases not led to end stages, such as fibrosis, ALT is higher than AST [13]. The mean of AST/ALT in both case and control groups was less than 1. This rules out alcohol consumption and also the relationship between alcohol consumption and enzymatic changes [1].

A statistically significant relation was seen between ALP and GGT increase and exposure to VCM which can show mild liver injury (cholestasis) due to VCM. Although such relation was not seen for other liver enzymes but their absolute values were higher in case group.

Previous studies have shown increase in liver transaminases in workers exposed to high levels of VCM [9,14-16]. Our study showed mild increase in liver enzymes with exposure to less than TLV concentrations of VCM. Considering the balance between case and control groups for factors such as age, sex, BMI, job experience and alcohol and tobacco consumption in our study, its results further approve the mentioned point.

There was a statistically significant relation between job experience in PVC production unit and increase in GGT and ALP which draws our attention to the importance of periodic exams and screening tests especially among those with higher job experiences.

Previous studies have shown contradictory results in the increase of liver enzyme levels in workers exposed to VCM [14,17,18].

In this study a statistically significant relation was found between ALP and GGT and VCM exposure which was in accordance with some previous studies [15]. Some studies have named GGT as a predictive indicator for liver problems in those exposed to VCM [19]. Due to low specificity this test does not have the necessary efficacy for those exposed to VCM when applied alone and is mostly efficient when used in combination with other liver tests [20].

For example it has been suggested that in the screening of VCM exposed workers at first ALP and indocyanine clearance test should be done to reduce the number of false positives and then GGT must be used to reduce the number of false negatives [1].

Assessment and discussion about biologic monitoring and early detection of VCM effects on liver is complicated. The most important urine metabolite of VCM is thiodiglycolic acid but it is of little value in biologic monitoring due to its non-specificity and that its concentration is not directly related to the concentration of VCM [1].

Different organizations have suggested different tests and biologic monitoring to detect the early effects of VCM on body (such as PFT and liver enzymes). In several studies the measurement of serum bile acids in workers exposed to VCM has been proposed as the best and most sensitive test for screening of VCM liver effects [21-23].

In some other studies hepatic and splenic sonography or platelet level was used to assess this effect and the VWF was used for the early detection of angiosarcoma [1,11,24-26].

On the whole, most studies have approved the suitability of measuring serum bile acids in the screening of VCM exposed workers. Unfortunately due to financial problems we didn't measure bile acid levels and didn't perform hepatic and splenic sonography.

It is important to say that workers at this PVC production unit have simultaneous exposure to other materials such as ethylene gas, but the measured levels of these materials were zero or negligible and they can not affect liver enzyme levels.

Liver tests performed in this study for cases were usually in the normal range and our findings in fact show the changes of these tests in working population. The clinical importance of these subtle changes of liver function in working populations is not clear and further longitudinal studies are necessary. Considering the results of this study, performing biochemical liver tests is recommended for screening and surveillance of workers exposed to low level VCM.

Naturally, liver transaminases and especially ALP and GGT are available, noninvasive and affordable tests for screening of workers exposed to VCM to detect early liver dysfunction.
